# Healthcare in Pakistan: Navigating Challenges and Building a Brighter Future

**DOI:** 10.7759/cureus.40218

**Published:** 2023-06-10

**Authors:** Quratulain Muhammad, Hadia Eiman, Faizan Fazal, Muhammad Ibrahim, Mudassar Fiaz Gondal

**Affiliations:** 1 Medicine, Holy Family Hospital, Rawalpindi, PAK; 2 Pediatric Surgery, Holy Family Hospital, Rawalpindi, PAK

**Keywords:** integrated healthcare, healthcare inequality, healthcare strategy, challenges in healthcare, global healthcare systems

## Abstract

A healthcare system is one of the most essential pillars of any country. The primary role of a healthcare system is to ensure that all people get the best available health facilities in a timely, acceptable, affordable, and accessible manner. However, for a healthcare system to function as such, it requires proper infrastructure and financial support. To a large extent, the healthcare system in Pakistan is facing several challenges. There is a massive shortage of hospitals, doctors, nurses, and paramedical staff. Most life-saving medications are too expensive for people to afford. Now and then, there is a shortage of medicines in the market. Above all, there exists a lack of trust in the healthcare system, which gives way to the ever-increasing quackery in the country. Two parallel systems exist in the healthcare system of Pakistan. One consists of public hospitals, and the other consists of private hospitals. The former is short even of basic healthcare facilities, and the latter is too costly for the people of Pakistan to afford. Solutions to the stumbling and compromised healthcare system of Pakistan are adequate financial support and infrastructure development. Stakeholders need to invest in the healthcare system; otherwise, the healthcare system in Pakistan will continue fighting for its survival rather than improving and competing with the healthcare systems of other nations in the region.

## Editorial

A healthcare system is defined as an organized aggregation of resources and personnel that work toward delivering healthcare services conforming to the demands, targets, and satisfaction of a country’s population. There are 195 countries globally, and all strive to promote health and reduce disease burden under the umbrella of global healthcare. There are four models currently used worldwide that bring together public and private facilities, providing a fitting rationale for the operation of healthcare systems [1].

Pakistan, a developing country, has kept the British healthcare system consisting of the “Beveridge model” in practice since the partition. Here, healthcare comprises a three-tier system of primary, secondary, and tertiary levels. The public and private sectors work together to provide the best possible care, but the healthcare burden, in general, has led to the failure of the deliverance of quality healthcare, more significantly in the government setup, which, in the last 10 years, has spent a meager 0.5-0.8% of gross domestic product (GDP) on healthcare, much lower than that recommended by the World Health Organization (WHO), which falls at 6% of the GDP [2].

In recent times, however, Pakistan has been identifying these gaps and formulating policies that guide toward the betterment of its healthcare. The Alma Ata Declaration directed the system toward providing primary healthcare (PHC). Since then, Pakistan has covered around 70% of the rural population, providing basic healthcare facilities, such as vaccination, mother and child healthcare services, and nutrition. Later advancements have led to the inclusion of individuals’ well-being, encompassing social and mental health measures along with physical health as determinants of the quality of life.

Pakistan, as a member of the United Nations (UN), strives to achieve the set benchmarks in healthcare by the year 2030. Many programs have been made functional to provide vertical and horizontal integration in healthcare. Currently around 17 in number, these programs have changed the architecture of healthcare in Pakistan to face much brighter horizons. Pakistan boasts as one of the pioneer countries in advocating public-private partnerships (PPPs), which has yielded fruitful outcomes. The National Tuberculosis (TB) Control Program, Child and Maternal Health Awareness Program, and Expanded Program on Immunization are working examples. The most revered initiative of the Lady Health Worker (LHW) Program ensures door-to-door delivery of PHC and has been a significant milestone in progressing toward the attainment of the utmost level of healthcare. Approximately 100,000 LHWs are presently working in five provinces of Pakistan. Each LHW covers a population of 1,000. Volunteering from the population and the awareness spread by these specifically trained females has been impactful.

The government has consistently put in the effort to reduce healthcare costs in hospitals and has established autonomous bodies to relegate a more efficient and effective healthcare delivery [3]. Pakistan has a long way ahead toward the development of an effective, accessible, and affordable healthcare system. The system is plagued with numerous flaws, ranging from inadequate infrastructure to inequitable distribution of healthcare facilities. The lack of adequate healthcare infrastructure is one of Pakistan’s biggest challenges. There is an extreme shortage of healthcare facilities, including hospitals, clinics, and diagnostic centers.

The chronic underfunding of the health sector is a massive reason for the lack of infrastructure, burdened by corruption, an unstable political system, and inequitable distribution of resources. Pakistan needs to construct and equip many more tertiary care and teaching centers. Currently, healthcare expenditure accounts for a mere 0.4% of Pakistan’s GDP, well below the WHO-recommended GDP to be spent on healthcare, i.e., 6% for low-income countries [2]. Moreover, this funding is inequitably distributed to Pakistan’s urban and developed cities. Hence, access to healthcare services is marked by stark disparities, with the rural population and low-income communities lacking basic healthcare facilities.

In 2020, the WHO published a health workforce profile of Pakistan, highlighting the country’s significant shortage of nurses [4]. Due to lacking incentives and cultural limitations, there is a shortage of trained nurses. Only 5% of nurses have an education of Bachelor of Science (BSc) or above, which is an alarming statistic for the healthcare system of Pakistan, as nurses and paramedical staff are the backbones of the country’s healthcare workforce [4]. Similarly, the doctor-to-patient ratio in Pakistan is 1:1300, significantly less than WHO’s recommended ratio of 1:1000 [2]. The workforce shortage can be attributed to a lack of incentives for healthcare professionals leading to a massive brain drain.

Pakistan desperately needs to increase its healthcare budget to improve its health infrastructure and overcome its workforce shortage. The government should prioritize constructing and upgrading healthcare facilities, particularly in rural areas. In addition, efforts must be made to explore innovative financing models to generate funds for healthcare. The development of PPPs is an example of such innovative models. Under the universal health coverage (UHC) initiative of the WHO, Pakistan launched the Sehat Sahulat Program (SSP) in 2015. This operated through a collaboration between the government of Pakistan and private insurance companies. Its main goal has been to provide free healthcare services to vulnerable and marginalized communities. While this program has largely been a success and made significant strides in improving healthcare accessibility, some challenges, like limited coverage and delays in reimbursement, hinder its effectiveness [5]. Nevertheless, this program can serve as the foundation for other PPPs.

In conclusion, Pakistan’s healthcare system faces significant challenges in providing effective and equitable healthcare to its citizens. However, these challenges can be overcome by strategic planning, the allocation of adequate funds, and the government’s keen interest in improving the current conditions. The political unrest in Pakistan has played a huge role as the rapid change in management and leadership interrupts the continuity of policies. Improving the health sector must be a priority regardless of government or regime changes. Prioritizing healthcare as a fundamental pillar of national development is crucial for Pakistan’s progress toward developing effective healthcare, which serves its citizens in all capacities. The deficiencies in the healthcare system of Pakistan have been identified numerous times throughout the decades. Now, it is imperative that policies be made and steps be taken by all stakeholders to minimize and address these deficiencies.
